# An Inverse Finite Element Method for Determining the Tissue Compressibility of Human Left Ventricular Wall during the Cardiac Cycle

**DOI:** 10.1371/journal.pone.0082703

**Published:** 2013-12-19

**Authors:** Abdallah I. Hassaballah, Mohsen A. Hassan, Azizi N. Mardi, Mohd Hamdi

**Affiliations:** 1 Department of Mechanical Engineering, Faculty of Engineering, University of Malaya, Kuala Lumpur, Malaysia; 2 Center of Advanced Manufacturing & Material processing, Faculty of Engineering, University of Malaya, Kuala Lumpur, Malaysia; 3 Department of Mechanical Engineering, Faculty of Engineering, Assiut University, Assiut, Egypt; University Hospital of Würzburg, Germany

## Abstract

The determination of the myocardium’s tissue properties is important in constructing functional finite element (FE) models of the human heart. To obtain accurate properties especially for functional modeling of a heart, tissue properties have to be determined in vivo. At present, there are only few in vivo methods that can be applied to characterize the internal myocardium tissue mechanics. This work introduced and evaluated an FE inverse method to determine the myocardial tissue compressibility. Specifically, it combined an inverse FE method with the experimentally-measured left ventricular (LV) internal cavity pressure and volume versus time curves. Results indicated that the FE inverse method showed good correlation between LV repolarization and the variations in the myocardium tissue bulk modulus K (K = 1/compressibility), as well as provided an ability to describe in vivo human myocardium material behavior. The myocardium bulk modulus can be effectively used as a diagnostic tool of the heart ejection fraction. The model developed is proved to be robust and efficient. It offers a new perspective and means to the study of living-myocardium tissue properties, as it shows the variation of the bulk modulus throughout the cardiac cycle.

## Introduction

One of the tools that can potentially lead to the early detection of human heart failures is the understanding of the mechanical behavior of the human left ventricle (LV) in normal and diseased states. This leads to continuous interest in the determination of the material properties of myocardium through the mechanical testing of excised strips of myocardium. These strips under prescribed homogeneous loading conditions give stress-strain relationships. Originally, these tests were done uniaxially but more recently, biaxial tests were also performed. Uniaxial test is used to define passive stress-strain relationships in the fiber direction [1]. It is very useful test in determining the general characteristics of the behavior of cardiac tissue in both healthy and diseased states, but is not sufficient to provide a unique description of three-dimensional (3D) constitutive behavior of the myocardium. Due to the incompressibility of cardiac tissue, biaxial tests can be used to determine certain multidimensional stress-strain relationships for the fiber and cross-fiber [2]. Despite the frequent assumption that human myocardial tissue is incompressible, the fact remains: all myocardial tissues have some degree of compressibility. Furthermore, the issue of the compressibility of myocardium tissue is raised due to the systolic intra- and extra-vascular blood displacements [3]. As a result of the previously mentioned fact, it is clear that the myocardium tissue compressibility changes during the cardiac cycle.

The information embodied in the myocardial tissue bulk modulus adds further insight to the mechanical nature of the soft tissue. Bulk modulus is very important as a standalone parameter, and as additional information to the shear/Young’s modulus. Precise values of the myocardium bulk modulus are especially required to improve the accuracy of finite element (FE) simulation for the modeling of the human heart. In the last several decades, publications related to cardiac modeling have handled the myocardial bulk modulus in many different approaches. The published values of myocardial bulk modulus recorded by researchers who were interested in simulating the performance of the LV during the diastolic phase are quite small, for instance 28 kPa [4] and 160 kPa [5]. This may be attributed to small changes in ventricular wall volume. Some studies evaluated the bulk modulus under the assumption that during the rapid filling phase, the volume change of the ventricular wall should be less than 10%. Additionally, relatively high values of bulk modulus were used by researchers who analyzed the systolic phase, for example 380 kPa [6], 600 kPa [7], and 25 MPa [8]. Using high values for bulk modulus during systolic phase are due to the systolic intra- and extra-vascular blood displacements that give rise to the compressibility of the tissue. Mean constant value of myocardial bulk modulus was assumed during each cardiac phase.

Despite the widespread use of uniaxial and biaxial tests for determining the myocardium characteristics, there are four major problems that arise from these kinds of studies [9], [10]:

1. Tests were carried out on non-human tissue, and as a result may not be directly applicable to humans.
2. The properties may not be directly applicable to FE models of the heart due to heterogeneous behavior of the myocardium.
3. The mechanical properties of myocardium changed drastically immediately after death.
4. The variation in the values of the mechanical properties according to the experimental loading conditions.

All the above limitations lead researchers to find different ways to run their experiments without having to excise samples of myocardium. Hence, a group of researchers moved to use Magnetic Resonance Imaging (MRI) and FE or mathematical methods to determine the mechanical properties *in vivo* [11], [12], while others applied the scanning acoustic microscope with high frequency ultrasound to measure the bulk modulus and describe the mechanical properties of the myocardium [13]. However, most bulk modulus experimental values obtained from these studies are very high (≈3 GPa), and cannot be used directly to FE modeling. To overcome these shortcomings, the FE approach is suggested to determine, *in vivo,* the myocardial tissue bulk modulus during the cardiac cycle.

Usually, FE analysis can be done directly once the input parameters such as LV geometry, LV internal cavity pressure and the myocardium tissue properties are known. Once the model is constructed, the desired outputs such as deformation behavior, LV cavity volume, LV wall stresses and strains can be predicted from the model. However, it is not uncommon in reality that some or all of the output values are known from experiments beforehand, while some of the input parameters still need to be determined. This requires the FE analysis to be done in an inverse way, where iteration of FE simulation is performed to find the material properties that give the best fit between the computed and experimentally measured LV internal cavity volumes. An inverse FE approach is a complex engineering process that can determine the unknown causes of known consequences. This approach has the advantage that the determination of the dynamic properties is measured non-invasively [14]–[17].

A novel method combining the experimentally measured LV pressure-volume curves and an inverse FE method is proposed to determine myocardial bulk modulus. The main purpose of this research is to develop an inverse FE procedure with ANSYS® computer code for the determination of the bulk modulus of human LV during cardiac cycle. The proposed inverse technique is based on published experimental measured LV pressure-volume curves. By using these outputs of published LV experimental data, the bulk modulus versus time curve is traced through inverse technique. Based on the obtained results, the repeated changes of myocardium tissue bulk modulus in the LV wall during cardiac cycle result in a highly efficient global function of the normal heart. Therefore, the myocardium bulk modulus can be effectively used as a diagnostic tool of the heart ejection fraction.

## Methods

### 2.1 Left Ventricle Geometry and FE Model

**Figure 1:**
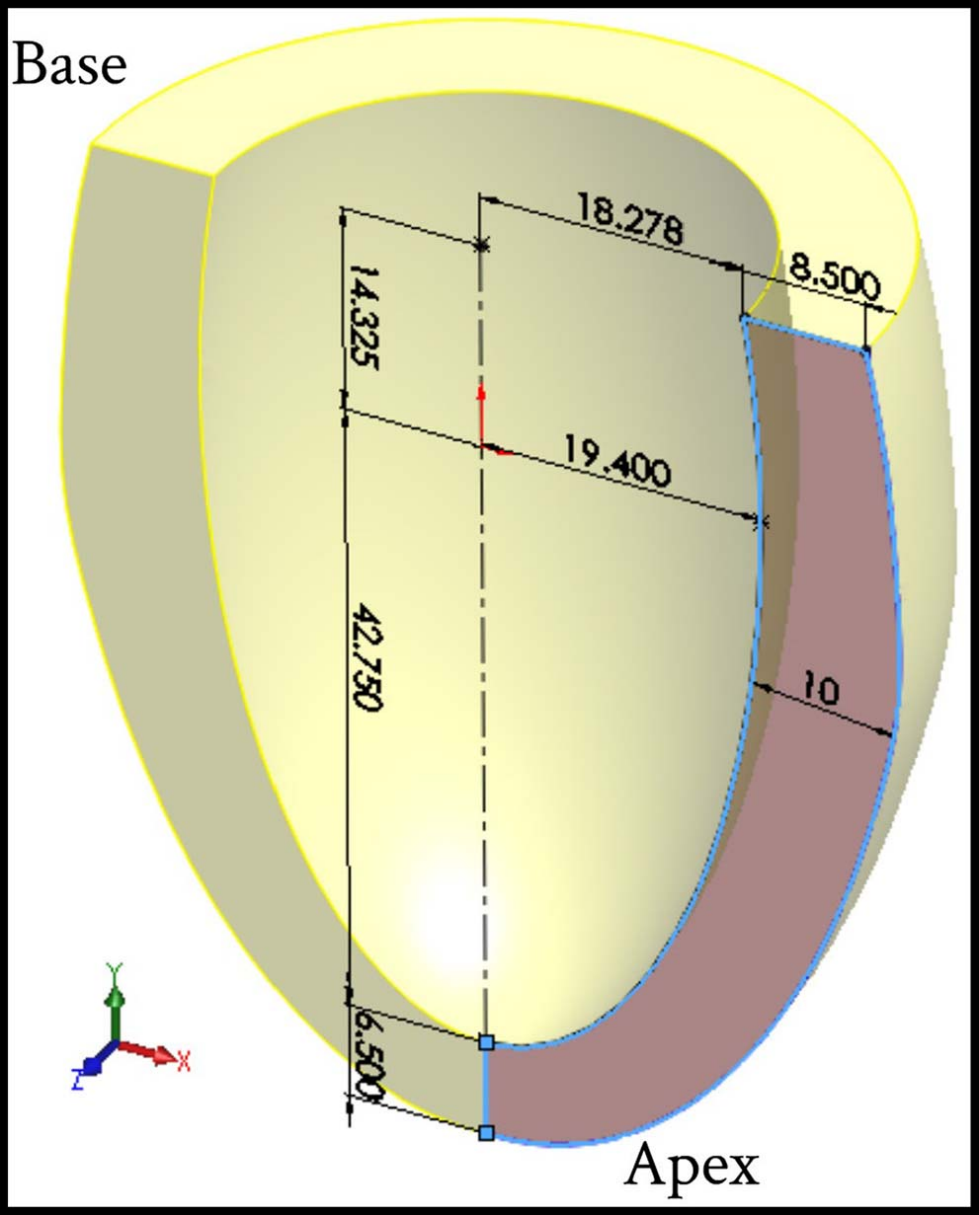
Geometric parameters of the thick-walled ellipsoid truncates at two thirds of major axis used to simulate LV model (for clarification half solid model presented).

**Figure 2:**
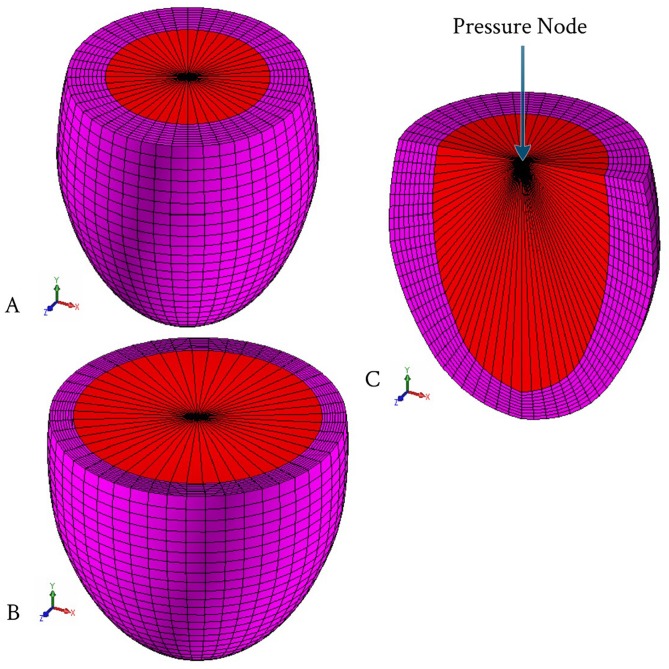
The left ventricular FE mesh. (A) Initial shape of the FE mesh; (B) Deformed shape of the FE mesh at the LV end-diastolic; and (C) Section view in the FE mesh to declare the elements used to simulated the LV cavity.

An inverse FE model was adopted to evaluate the LV tissue compressibility during cardiac cycle. 3D-FE model was built to simulate the deformation mechanics of the LV using ANSYS® commercial software. To simplify the analysis, FE simulation model was represented by an ellipsoid, truncated at two-thirds of the major axis including two sets of fibers (myocardial fibers bound by a mesh of collagen fibers), which were attached to each other to form a spatial network. The geometric parameters and dimensions of the LV model in the initial undeformed configuration (at hypothetical zero pressure applied inside the LV cavity) are shown in Figure 1. The wall thickness of the LV model, in the reference unstressed state, was divided into seven equal thickness layers. Figure 2A shows the initial shape of a typical FE mesh used for the present computations, while Figure 2B shows the end-diastolic deformed shape of the FE mesh. The wall of LV model was discretized with 20-node hexahedral element; with the exception of the apical region which was meshed using 10-node tetrahedral element. The current FE mesh consisted of 22,080 total number of elements and 29,777 nodes. This discretization of the current LV model was sufficient and any further mesh refinement showed very little improvement [18]. The LV blood cavity was modeled by the hydrostatic fluid 3D solid element; this element is well suited for calculating fluid (blood) volume and pressure for coupled problems involving fluid-solid interaction. Hydrostatic fluid elements were overlaid on the faces of 3D solid element enclosing the fluid volume. Figure 2C shows the section view in the FE mesh in order to clarify the shape of the elements used for modeling the LV internal cavity. The hydrostatic fluid element was defined by nine nodes; eight nodes on the internal surface of LV cavity (endocardium) and the remaining ninth node at the base center, which is also called “the pressure node”. This pressure node was used to define the LV pressure which was assumed to be uniform through the LV cavity; the predefined value of pressure was automatically moved to the centroid of the fluid volume. In all FE computations the LV cavity and LV wall volumes were kept constant at 50 ml and 73.6 ml respectively. The circumference of the LV internal cavity was divided into 48 equally spaced divisions, i.e. 48 elements along the circumferential direction.

Two separate parallel sets of 3D fiber network; contractile muscle fibers bundles (myofibers) bound by a mesh of collagen fibers were embedded within continuum 3D solid element to reproduce the globally anisotropic behavior of cardiac tissue. Computationally, these fibers were modeled as layers of uniformly spaced reinforcement bars (rebar) within the continuum 3D elements; each layer was set to be parallel to two of the isoparametric directions in the element’s local coordinate system.

The collagen fibers were arranged in the radial direction, while the myofibers orientation changed with position within the LV wall. The 3D reinforcing element was used to simulate both myofiber and collagen fiber. The continuum 3D element is suitable for simulating reinforcing fibers with arbitrary orientations and used to model the myofiber force. The force was restricted in the direction of the fiber only (uniaxial fiber tension). The reinforcing element was firmly attached to its base element, i.e. no relative movement between the reinforcing element and the base was allowed. FE computations were conducted with myofibers and collagen fibers volume fractions of 0.7 and 0.015, respectively [19], [20].

### 2.2 LV Myofiber Architecture

**Figure 3:**
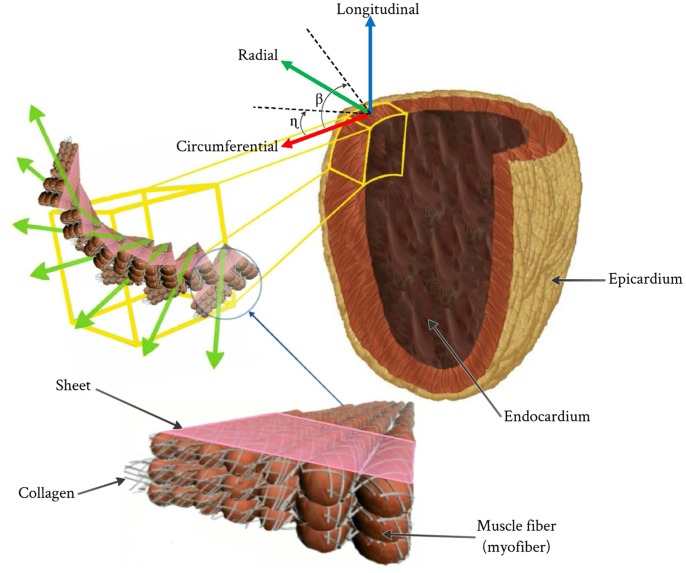
Resprentation of fibers in the LV and the local myocardial coordinate system.

**Figure 4:**
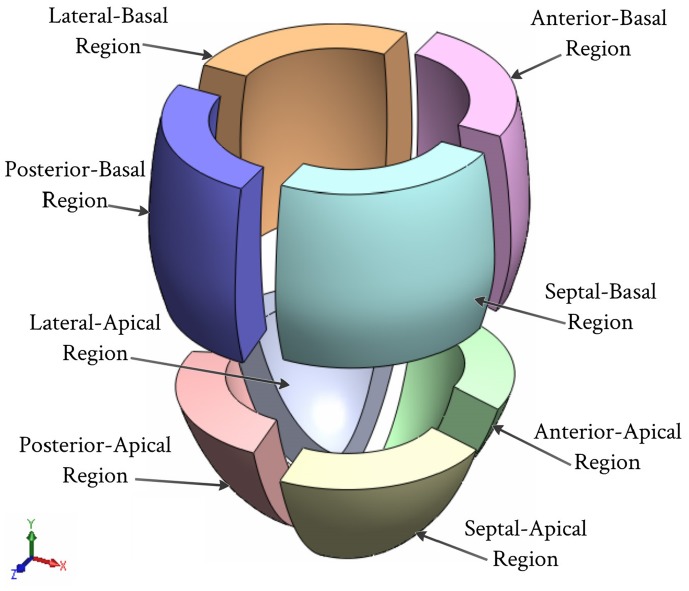
Eight regions of the LV that are used for clarification the helix angle (β).

The human muscle fibers oriented at different angles throughout the ventricle wall in the form of sheets that are separated by a complex structure of cleavage surfaces (see Figure 3) [21]–[23]. The myofibers could be fully described by two inclination projection angles; the helix angle (β) and the transverse angle (η) in the two perpendicular planes. The transmural distribution of helix angle (β) varies in a linear manner through wall thickness from the lowest negative value at the epicardium to the highest positive value at the endocardium, while the transverse angle (η) varies in a linear manner through along the longitudinal axis of the LV from the lowest negative value at the apex to the highest positive value at the base [24], [25], 21,26,27. The corresponding variations of the fiber helix angles (β) through the eight regions are as follows (see Figure 4); septum-basal region (−60° : +40°), anterior-basal region (−40° : +60°), lateral-basal region (−20° : +50°), posterior-basal region (−20° : +60°), septum-apical region (−50° : +40°), anterior-apical region (−20° : +60°), lateral-apical region (−20° : +50°), and posterior-apical region (−20° : +60°) vary smoothly across the LV wall thickness from a negative angle at epicardium to positive angle at endocardium respectively [28].

The distribution of transverse angle (η) was taken as a variable value in a linear manner from +15° at the base to the circumferential direction (η = 0°) in the equatorial region to −15° at the apex [24]. Meanwhile, the collagen fibers were arranged in the radial directions.

### 2.3 Loading and Boundary Conditions

**Figure 5:**
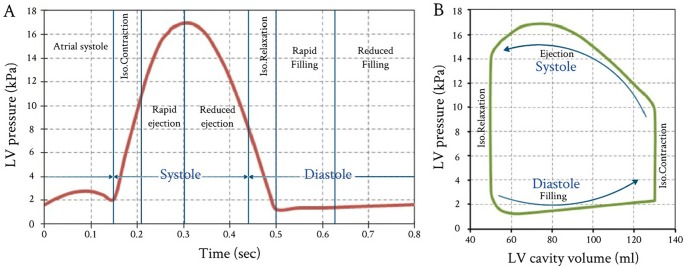
Variations of LV pressure and volume vs. time throughout one cardiac cycle. (A) Measured LV pressures started from atrial systole; and (B) Accompanied PVR loop with stroke volume (SV) 80 ml, LV peak pressure 16.93 kPa, and ejection fraction (E_f)61.55%. (The LV pressure data obtained from published measurements by Hall, 2011 [29]).

To demonstrate the performance of the proposed FE model, LV pressure versus time curve for a healthy human heart was used (see Figure 5). This case was adapted from the experimental measurements carried out by Hall [29]. The LV pressure versus time curve given in Figure 5 represents the FE model with applied loads, while the accompanying LV internal cavity volume were adopted as the FE model target values.

The external surface of the heart is affected by the surrounding organs (lungs, ribcage and diaphragm). In order to simulate the boundary conditions imposed by these surrounding organs and tissues, an elastic foundation 3D structural surface effect element with a stiffness *K_f_ = *0.02 kPa was used [4], [30]. Due to the lack of information about the influence of the surrounding organs and tissue on the deformation of the heart, a uniform elastic foundation was assumed.

To prevent rigid body motion of the model, degrees of freedom for all nodes at the base were suppressed in the longitudinal direction (UY = 0). To avoid possible excessive deformations of FE mesh elements, the pressure node was fixed laterally (UX = UZ = 0) (see Figure 2).

### 2.4 Myocardium Active and Passive Material Properties

The LV pressure response to its changing volume during ejection phase relies on the active elastance property activated by the muscle action. During systolic phase, the muscle generate adequate contractile force (muscle active force) to provide sufficient LV pressure to open the aortic valve, and pump an appropriate volume of blood [31]. At the beginning of the isovolumic contraction, the LV internal cavity pressure increases rapidly until the peak pressure and consequently the muscle contraction force continually increase, to sustain increasing LV pressures. Both the LV pressure and muscle contraction force increased simultaneously until its peak. During cardiac cycle the LV wall is subjected to dual forces; the active force generated by the myocardium muscles and the force generated by the blood pressure in the LV cavity. The active muscle force is not only governed by the myocardium passive properties but also depend mainly on myocardium active elastance operating throughout the cardiac cycle.

**Figure 6:**
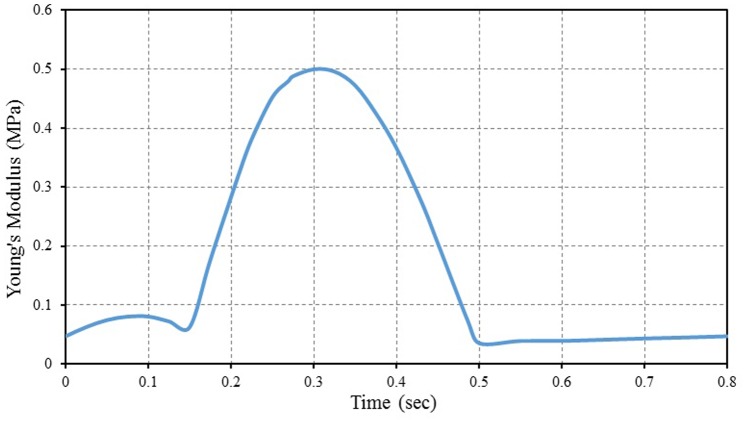
Calculated active myofiber Young’s modulus (myocardial stiffness) during one cardiac cycle.

In this study we suggest a simple method to calculate the active myofiber elastance properties by taking into consideration that the maximum value of myofiber Young’s modulus does not exceed 0.5 MPa [32], [33]. The active myofiber elastic properties during cardiac cycle can be simply calculated by multiplying the LV pressure value (given in Figure 5) with constant value that equals to 29.5, i.e. the value of active myofiber Young’s modulus depending linearly on the intracavital pressure. This constant value is equal to 0.5 (maximum myofiber Young’s modulus) divided by the maximum value of LV pressure. The computation of active myofiber Young’s modulus based on the above calculated constant represents the hypothesis of the present study. Figure 6 shows the calculated active myofiber Young’s modulus versus time during one cardiac cycle. The time dependent calculated values of active myofiber Young’s modulus is applied on the FE model in order to calculate the contraction force in the myocardial wall.

The myocardium tissue (matrix) was represented as an isotropic, slightly compressible hyperelastic material with relatively soft properties. The Ogden model with two parameters was used in the present study and having the following parameters; µ_1_ = 0.22 MPa, µ_2_ = 0.11 MPa, α_1_ = 11.77 and α_2_ = 14.34 [34]. The tissue bulk modulus K of the LV model was tuned so that the simulation behaves in accordance with the measured pressure vs. volume curve (patient-specific datasets).

The collagen fiber behavior was represented by isotropic linear elastic with large displacements, to simulate the large strains occurring in the collagen fiber during LV filling. Collagen properties are as follows; the Young’s modulus (E) = 50 kPa, Poisson’s ratio (**ν**) = 0.49, and density (**ρ**) = 1000 kg/m^3^ [35].

### 2.5 Solution Procedures and Inverse Identification of Myocardium Tissue Compressibility

**Figure 7:**
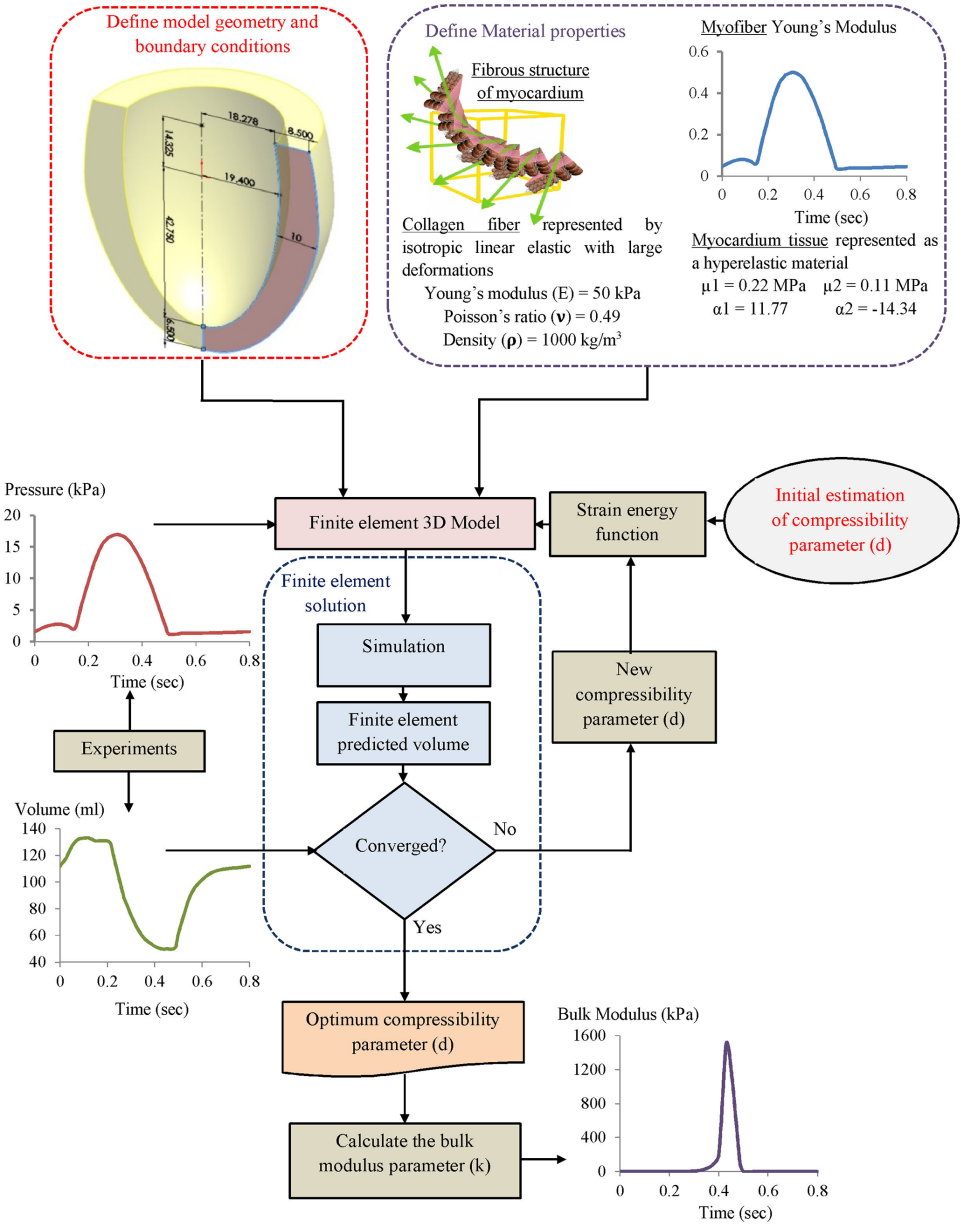
Flowchart of the inverse FE computation sequences for the LV tissue compressibility identification.

Figure 7 shows the inverse FE computation sequence procedures for the evaluation of the myocardial bulk modulus. The myocardium tissue bulk modulus satisfying the required output was inversely identified. The LV pressure versus time curve (see Figure 5) as the inputs were applied on the internal surface of LV cavity (endocardium) via the pressure node of the hydrostatic fluid element (see Figure 2C). The other passive material properties of myocardium tissue (see item 2.4) were fixed as material constants. The calculated active myofiber Young’s modulus (see Figure 6) was used to simulate the muscle active contraction force generated through the LV wall during the cardiac cycle.

An initial guess value of tissue compressibility for the myocardium was then applied and by successive computations this was refined until the calculated LV cavity volume matched the measured volume. Iterative FE computation for the LV cavity volume during the cardiac cycle was carried out. At the end of each computation step, the predicted LV cavity volume of the FE model was compared to the measured value. The computation ended if the relative error between the computed and measured values ≤1%. For this calculation, the total computation time for each run was taken about 1000 s using PC Intel Core i7 (2.93 GHz with RAM 2.00 GB). At the start of all FE computations the LV wall initially was assumed to be stress free (LV cavity pressure equals zero).

### 2.6 FE Model Merits and Limitations

A new FE inverse model is presented that features the ability to determine the myocardial tissue bulk modulus during cardiac cycle. Other advantages of the proposed method include:

1. Slightly compressible hyperelastic tissue properties.
2. Realistic boundary conditions based on MRI observations.
3. Myofibers orientation simulated based on the obtained data from MRI.
4. The effect of interaction between blood and the internal cavity of LV wall.
5. The effect of surrounding organs and tissues on the deformation of the heart.

However, this model is limited in a number of important ways, including:

1. Simplified geometry for the LV.
2. An incomplete understanding of some heart diseases.
3. The model’s inability to study effects of electrical activation, blood flow, porous medium, and cardiac metabolism.
4. The used measured data taken for a healthy human heart “ideal proband”.
5. More realistic tissue mechanical properties of LV are still needed.

These limitations and weaknesses can be used as the bases in future model improvements.

## Results and Discussion

### 3.1 Volume Variations of LV Cavity During One Cardiac Cycle

**Figure 8:**
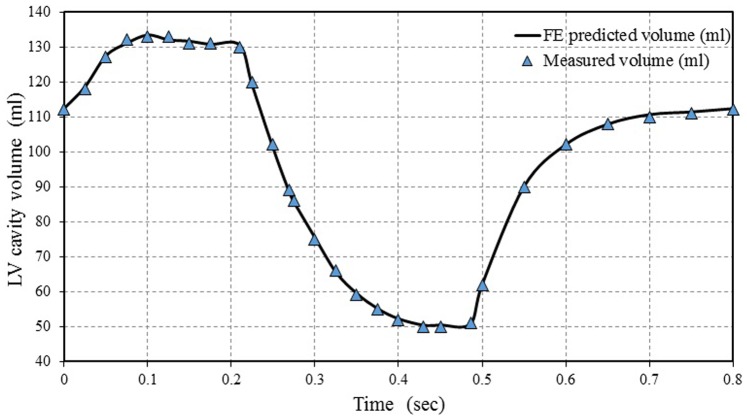
Comparison between the FE predicted LV cavity volume and experimentally measured data.

Figure 8 shows the comparison between the predicted FE and experimentally measured LV cavity volumes. The LV cavity volumes increased rapidly from 110 ml to 130 ml (end diastolic volume EDV = 130 ml), shortly after the beginning of atrial systole phase through time = 0.1 sec. Then, LV cavity volume remained constant during the isovolumic contraction phase until time = 0.21 sec. After that, a sudden decrease in LV cavity volume can be seen at the onset of the rapid ejection phase until time = 0.3 sec, followed by a slight decrease during the reduced ejection phase until time = 0.43 sec, at the end systolic phase. The size of LV cavity volume at the moment was equal to that at the end systolic volume (ESV = 50 ml). The LV cavity volume remained constant during the isovolumic relaxation phase until time = 0.5 sec, followed by a rapid increase during the rapid filling phase until time of 0.65 sec. Finally, the LV volume slightly increased during the reduced filling phase up to the end of cardiac cycle.

The LV cavity volume was locally adapted in response to several stimuli, such as systole phase (isovolumic contraction and ejection) and diastole phase (isovolumic relaxation, rapid filling, reduced filling and atrial contraction). Comparing the present simulation results of LV cavity volumes with corresponding values reported in groups of normal healthy subjects in previous studies [36]–[39], it was found that the present model can accurately predict the change in the LV cavity volume during the cardiac cycle. It can also be found that the various events (cardiac cycle phases) can be sharply defined. It should be noted that most of those studies ignored the effect of atrial systole on LV volume while others failed to accurately represent all cardiac cycle phases precisely isovolumetric contraction and isovolumetric relaxation.

### 3.2 Variations of Tissue Compressibility during One Cardiac Cycle

During cardiac cycle, the myocardium wall tissue is exposed to successive active contraction and relaxation in consequence of depolarization and repolarization, respectively. Due to heart beating and LV pressure dynamics response, a significant amount of energy can be expended to compress the heart wall, essentially squeezing the myocardium cells closer together.

**Figure 9:**
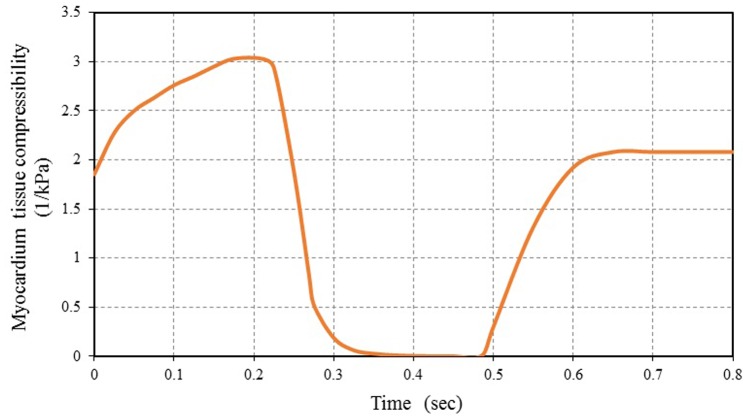
FE computed myocardial tissue compressibility during one cardiac cycle vs. time.

Figure 9 shows the FE results for the myocardium tissue compressibility variations during one cardiac cycle. It can be noticed that the myocardium tissue is nearly incompressible during a short period of time about 0.16 sec throughout the cardiac cycle (approximately not exceeding 20% of total cardiac cycle time). The tissue showed incompressible behavior during the reduced ejection and isovolumic relaxation phases. The myocardium tissue incompressibility is a common notion used in numerical simulations and analytical studies [40]–[43]. This is mainly for the purpose of simplifying the analytical formulations and the interpretation of experimental data.

From Figure 9, it can be noticed that myocardium tissue compressibility decreased rapidly at the beginning of the rapid ejection phase. After that, myocardium tissue compressibility increased gradually at the rapid filling phase and remained nearly constant during reduced filling, followed by further increase during the atrial systole (increased about 30% of the maximum of tissue compressibility). Increasing of myocardium tissue compressibility leads to LV wall growth and remodeling. The ability of the present FE model to simulate the observations of growth and remodeling consistent with the previous theoretical study obtained by Kroon et al. [40] and also with the empirical observations using diffusion tensor MRI carried out by Teske et al. [44]. The difference between the maximum myocardium tissue compressibility is 3 kPa^−1^ and its minimum value (≈ 0 kPa^−1^) can be used as a good index for the normal ventricular function. The decrease in this value means abnormal cardiac function occurrences, maybe due to myocardial infarction or heart dysfunction. The myocardium tissue compressibility increased with decreasing in the contractile force of the ventricles. This lead to an increase in LV cavity size which means that the heart cannot pump blood efficiently and structural alterations of the myocardium (i.e. heart is enlarged and the heart’s pumping ability is impaired). Hence, the myocardial tissue compressibility should be considered if myocardial performance, myocardial deformation and heart wall stresses, response time, is critical.

Like many biological tissues, myocardium tissue is able to adapt to changes in mechanical load through growth (change in mass) and remodeling (change in tissue properties) [40]. This hypothesis has confirmed by the results obtained using the present FE model. The LV wall volume was locally adapted (change in tissue compressibility) in response to several stimuli, such as early systolic fiber stretch, fiber shortening during ejection, and contractility.

### 3.3 Variations of Bulk Modulus during One Cardiac Cycle

**Figure 10:**
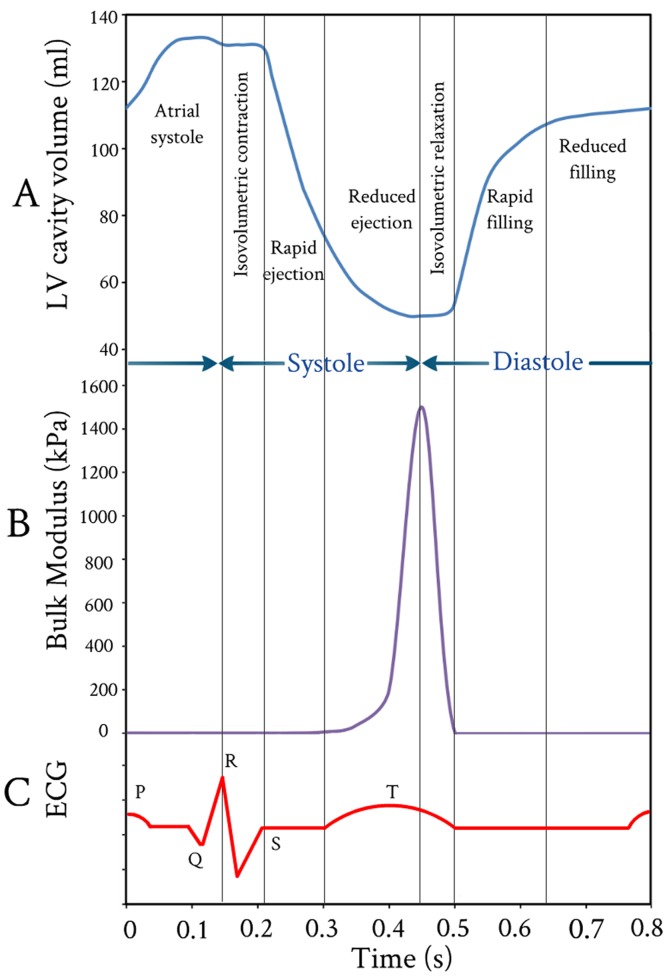
Variations of LV cavity volume, tissue bulk’s modulus, and electrocardiogram vs. time throughout one cardiac cycle. (A) FE computed LV cavity ventricular volume; (B) FE computed myocardium tissue bulk’s modulus; and (C) Accompanied electrocardiogram (ECG).

Figure 10B shows the FE results for the variations of myocardium bulk modulus during one cardiac cycle. Great variations in the values of the myocardium bulk modulus occurred during the rapid ejection and isovolumic relaxation phases. The myocardium bulk modulus reached to its maximum value at end of ejection and began to decline at the beginning of isovolumic relaxation, i.e. the tissue of LV wall was stiffened by contraction and softened by relaxation. It can be noticed that the myocardium bulk modulus increased exponentially during ejection phase till its peak value, then followed by a linear decrease during isovolumic relaxation phase (i.e. the myofibrils return to their original length). The peak value of bulk modulus occurred at 0.43 sec and the duration time for bulk modulus changes from 0.3 sec to 0.5 sec.

With regard to the timing, the computed duration times for bulk modulus changes were compared to the electrocardiogram (ECG) during one cardiac cycle (see Figure 10C). It can be noticed that the duration times for the onset and ending of LV repolarization marked by T wave (on surface ECG) agree very well with the onset and ending duration times of bulk modulus changes (see Figure 10C). Also, it can be noticed that the onset and ending times of LV repolarization from 0.3 sec to 0.5 sec are sharply defined. Actually, the electrical signals were not included in the present FE analysis, but their impacts are included in the present FE model by introducing the myofiber active elastance. From Figure 10A–C, it can be noticed that there is a good correlation (synchronization) between the instantaneous variation of myocardium tissue bulk modulus and the onset and ending of LV repolarization (T wave).

The bulk modulus for myocardium tissue predicted by the current FE model is considerably less than the experimental values measured by Masugata et al. [45]. This is potentially a result of the bulk modulus for myocardium tissue changed drastically immediately after death. Also, there are challenges in the proper acquisition of human myocardium tissue samples and protocols for appropriate experimentation. Diversity of results can also be explained by the differences in the FE models simulation and experimental conditions or the choice of model parameters.

In conclusion, it was concluded that cyclic variation of bulk modulus as predicted by FE model exists during the cardiac cycle with the myocardium tissue being stiffer in systole than it is in diastole. Such behaviors was observed in the experimentally measured shear stiffness for normal myocardium throughout the cardiac cycle using Magnetic Resonance Elastography (MRE) by Kolipaka et al. [46].

### 3.4 Comparison between Predicted FE Bulk Modulus and Ejection Fraction

**Figure 11:**
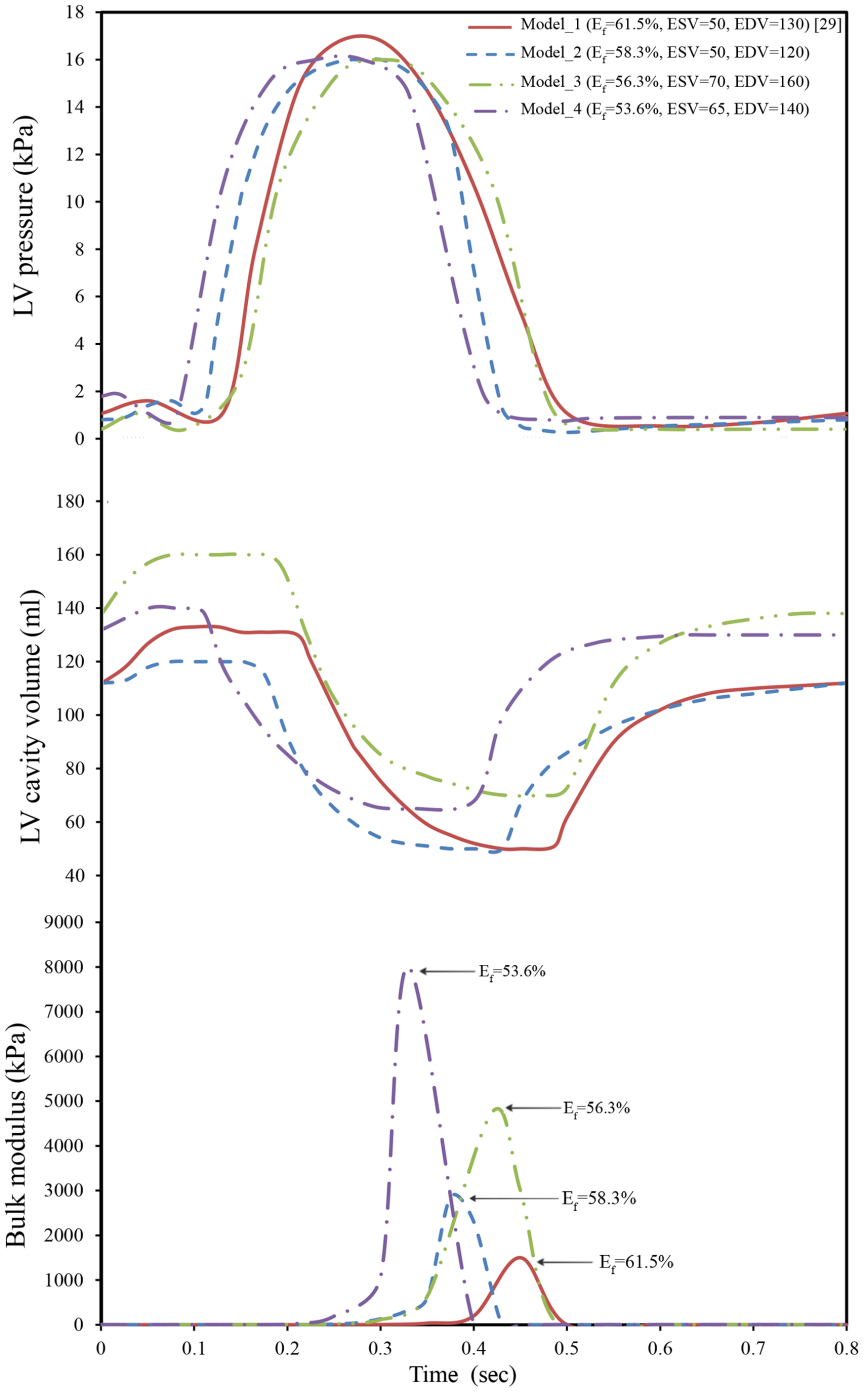
Variations of LV pressure, volume, and tissue bulk’s modulus vs. time through one cardiac cycle. (A) Measured LV pressures for different cardiac cycles; (B) Measured LV cavity volumes; and (C) FE computed myocardium tissue bulk’s modulus.

The predicted myocardium bulk modulus (K) and ejection fraction (E_f_) were compared. Four different LV pressure-volume diagrams with different sets of physiological conditions were used, see Figure 11A, B. The initial LV cavity volumes of the used models were 50 ml, 50 ml, 65 ml, and 70 ml for Model_1, Model_2, Model_3, and Model_4 respectively, while the LV wall thickness was kept constant as described in Figure 1. Figure 11C shows the variations of the predicted myocardium bulk modulus during one cardiac cycle. It can noticed that a discrepancy among the peak values of myocardium bulk moduli exists; 1500 kPa, 2855 kPa, 4760 kPa, and 8000 kPa, which correspond ejection fractions of 61.5% [29], 58.3% [47], 56.3% [48] and 53.6% [49] respectively.

**Figure 12:**
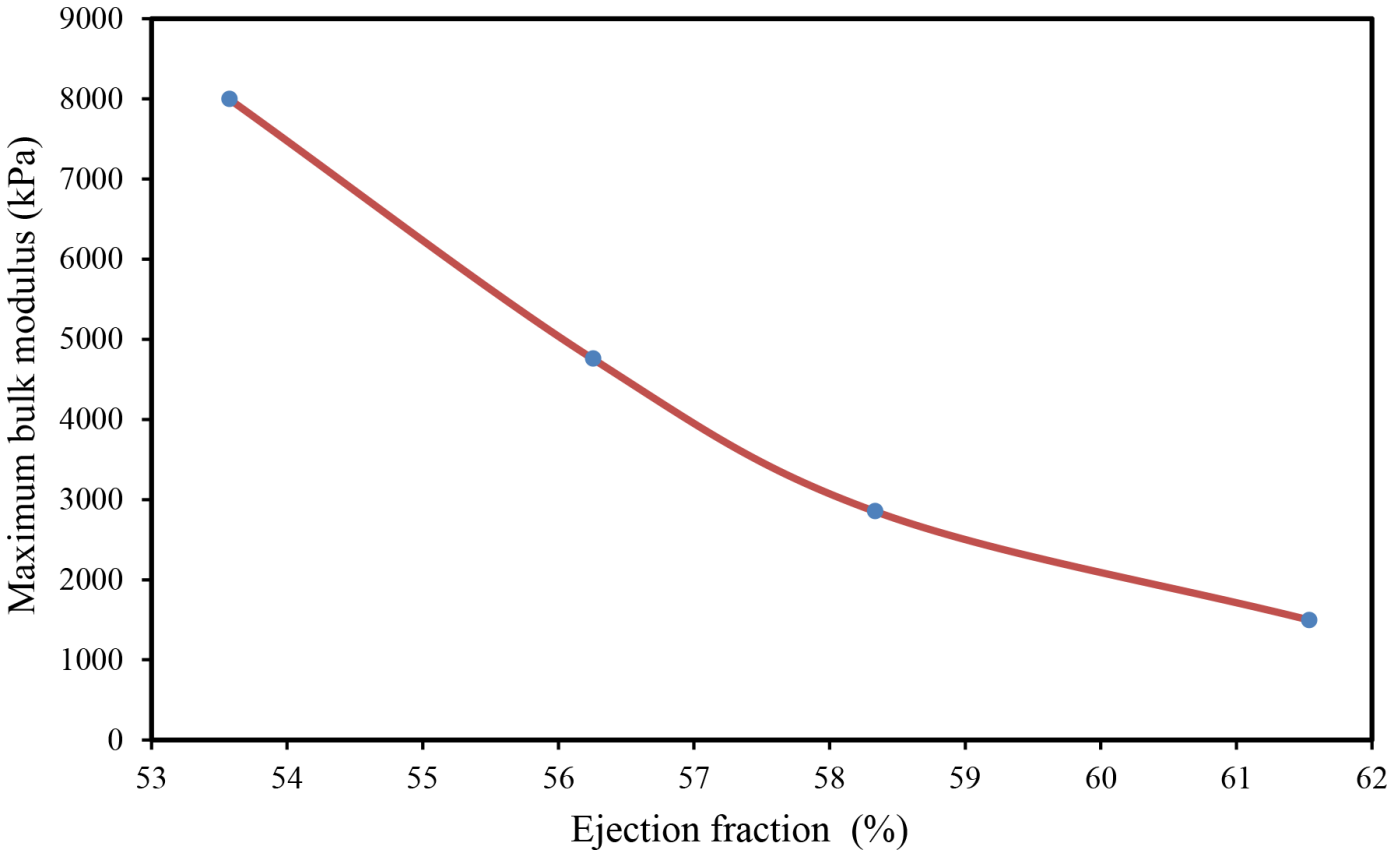
Comparison between the FE predicted maximum bulk modulus and ejection fraction.

Figure 12 shows the variations of the maximum bulk modulus versus ejection fraction. It is clear that the ejection fraction increased with decreasing peak values of myocardium bulk modulus. Such decrease (i.e. increases of myocardial tissue compressibility) caused an increase of myocardial contraction, which led to an increase of heart ejection fraction. Further study is still required to verify the correlation among the myocardium tissue bulk modulus as a marker for heart function, strain and strain rate. Finally, the above results confirm the hypothesis, in the computing the active myofiber Young’s modulus and the usage of inverse FE analysis for the determination of myocardium tissue bulk modulus.

## Conclusions

A novel method combining FE inverse model and LV pressure-volume diagrams was developed to determine the myocardial tissue bulk modulus. The human LV wall (for simplicity) modeled as a thick-walled ellipsoid truncated at two thirds of major axis with spatial myofiber angle distribution was used. The ellipsoidal geometry has been chosen to model the human LV, as it is close to the real anatomical shape, and yet quite simple. The myocardial bulk modulus of different LV pressure-volume diagrams (with four different sets of physiological conditions; stroke volume, LV maximum pressure and ejection fraction), was determined. Based on the results and discussion presented in the preceding section, the following conclusions can be drawn:

1. The myocardium bulk modulus can be used as a diagnostic tool (clinical indicator) of the heart ejection fraction.
2. Our results show that the present FE model is sensitive to the overall cardiac function parameters expressed in terms of LV pressure-volume variations during cardiac cycle and ejection fraction.
3. The calculations of active myofiber Young’s modulus (myocardium active properties) based on the LV pressure proved their correctness.

With this research, the authors would like to recommend further investigations on the subject of compressibility of myocardium tissue, which is still debated and remains a challenging experimental topic.

## References

[1] Kohl P, Sachs F, Franz MR (2011) Cardiac mechano-electric coupling and arrhythmias: OUP Oxford.

[2] Fung Y, Cowin S (1994) Biomechanics: Mechanical properties of living tissues. Journal of Applied Mechanics 61: 1007–1007.

[3] Yin F, Chan C, Judd RM (1996) Compressibility of perfused passive myocardium. American Journal of Physiology-Heart and Circulatory Physiology 271: H1864–H1870. 10.1152/ajpheart.1996.271.5.H1864 8945902

[4] Bettendorff-Bakman DE, Schmid P, Lunkenheimer P, Niederer P (2006) A finite element study relating to the rapid filling phase of the human ventricles. J Theor Biol 238: 303–316. 16002095 10.1016/j.jtbi.2005.05.009

[5] Veress AI, Gullberg GT, Weiss JA (2005) Measurement of strain in the left ventricle during diastole with cine-MRI and deformable image registration. 10.1115/1.2073677 16502662

[6] Shim J, Grosberg A, Nawroth JC, Kit Parker K, Bertoldi K (2012) Modeling of cardiac muscle thin films: Pre-stretch, passive and active behavior. J Biomech 45: 832–841. 22236531 10.1016/j.jbiomech.2011.11.024 PMC3294204

[7] Dorri F, Niederer P, Lunkenheimer P (2006) A finite element model of the human left ventricular systole. Computer Methods in Biomechanics and Biomedical Engineering 9: 319–341. 17132618 10.1080/10255840600960546

[8] Marchesseau S, Delingette H, Sermesant M, Sorine M, Rhode K, et al.. (2012) Preliminary specificity study of the Bestel-Clément-Sorine electromechanical model of the heart using parameter calibration from medical images. Journal of the Mechanical Behavior of Biomedical Materials. 10.1016/j.jmbbm.2012.11.021 23499249

[9] Yettram A, Beecham M (1998) An analytical method for the determination of along-fibre to cross-fibre elastic modulus ratio in ventricular myocardium–a feasibility study. Medical engineering & physics 20: 103–108. 9679228 10.1016/s1350-4533(98)00009-5

[10] Périé D, Dahdah N, Foudis A, Curnier D (2013) Multi-parametric MRI as an indirect evaluation tool of the mechanical properties of in-vitro cardiac tissues. BMC cardiovascular disorders 13: 1–9. 23537250 10.1186/1471-2261-13-24 PMC3617013

[11] Augenstein KF, Cowan BR, LeGrice IJ, Young AA (2006) Estimation of cardiac hyperelastic material properties from MRI tissue tagging and diffusion tensor imaging. Medical Image Computing and Computer-Assisted Intervention–MICCAI 2006: Springer. 628–635. 10.1007/11866565_77 17354943

[12] Wang VY, Lam H, Ennis DB, Cowan BR, Young AA, et al (2009) Modelling passive diastolic mechanics with quantitative MRI of cardiac structure and function. Med Image Anal 13: 773. 19664952 10.1016/j.media.2009.07.006 PMC6467494

[13] Dent CL, Scott MJ, Wickline SA, Hall CS (2000) High-frequency ultrasound for quantitative characterization of myocardial edema. Ultrasound in medicine & biology 26: 375–384. 10773367 10.1016/s0301-5629(99)00144-1

[14] Balaraman K, Mukherjee S, Chawla A, Malhotra R (2006) Inverse Finite Element Characterization of Soft Tissues Using Impact Experiments and Taguchi Methods. SAE Paper: 01–0252.

[15] Zenker S, Rubin J, Clermont G (2007) From Inverse Problems in Mathematical Physiology to Quantitative Differential Diagnoses. PLoS Comput Biol 3: e204. 17997590 10.1371/journal.pcbi.0030204 PMC2065888

[16] Xu Z-H, Yang Y, Huang P, Li X (2010) Determination of interfacial properties of thermal barrier coatings by shear test and inverse finite element method. Acta Materialia 58: 5972–5979.

[17] Evans S, Avril S (2012) Editorial: Identification of material parameters through inverse finite element modelling. Computer Methods in Biomechanics and Biomedical Engineering 15: 1–2. 10.1080/10255842.2012.650321 22229516

[18] Hassaballah AIM, Hassan MA, Mardi NA, Hamdi MA (2013) Modeling the effects of myocardial fiber architecture and material properties on the left ventricle mechanics during rapid filling phase. Journal of applied mathematics and information sciences; in press.

[19] LeGrice IJ, Smaill B, Chai L, Edgar S, Gavin J, et al (1995) Laminar structure of the heart: ventricular myocyte arrangement and connective tissue architecture in the dog. American Journal of Physiology-Heart and Circulatory Physiology 269: H571–H582. 10.1152/ajpheart.1995.269.2.H571 7653621

[20] Stevens C, Remme E, LeGrice I, Hunter P (2003) Ventricular mechanics in diastole: material parameter sensitivity. J Biomech 36: 737–748. 12695004 10.1016/s0021-9290(02)00452-9

[21] Helm P, Beg MF, Miller MI, Winslow RL (2005) Measuring and mapping cardiac fiber and laminar architecture using diffusion tensor MR imaging. Ann Ny Acad Sci 1047: 296–307. 16093505 10.1196/annals.1341.026

[22] Sengupta PP, Korinek J, Belohlavek M, Narula J, Vannan MA, et al (2006) Left ventricular structure and function: basic science for cardiac imaging. J Am Coll Cardiol 48: 1988–2001. 17112989 10.1016/j.jacc.2006.08.030

[23] Arts T, Lumens J, Kroon W, Delhaas T (2012) Control of Whole Heart Geometry by Intramyocardial Mechano-Feedback: A Model Study. PLoS Comput Biol 8: e1002369. 22346742 10.1371/journal.pcbi.1002369 PMC3276542

[24] Kerckhoffs R, Bovendeerd P, Kotte J, Prinzen F, Smits K, et al (2003) Homogeneity of cardiac contraction despite physiological asynchrony of depolarization: a model study. Ann Biomed Eng 31: 536–547. 12757198 10.1114/1.1566447

[25] Chen J, Liu W, Zhang H, Lacy L, Yang X, et al (2005) Regional ventricular wall thickening reflects changes in cardiac fiber and sheet structure during contraction: quantification with diffusion tensor MRI. American Journal of Physiology-Heart and Circulatory Physiology 289: H1898–H1907. 16219812 10.1152/ajpheart.00041.2005

[26] Lombaert H, Peyrat JM, Croisille P, Rapacchi S, Fanton L, et al (2011) Statistical Analysis of the Human Cardiac Fiber Architecture from DT-MRI. Lect Notes Comput Sc 6666: 171–179.

[27] Lombaert H, Peyrat JM, Croisille P, Rapacchi S, Fanton L, et al (2012) Human Atlas of the Cardiac Fiber Architecture: Study on a Healthy Population. Ieee T Med Imaging 31: 1436–1447. 10.1109/TMI.2012.2192743 22481815

[28] Rohmer D, Sitek A, Gullberg GT (2006) Reconstruction and visualization of fiber and laminar structure in the normal human heart from ex vivo DTMRI data. Tech. Rep., Lawrence Berkeley National Laboratory. 10.1097/RLI.0b013e3181238330 18030201

[29] Hall JE (2011) Guyton and Hall Textbook of Medical Physiology: Enhanced E-book: Saunders.

[30] Bettendorff-Bakman DE, Schmid P, Lunkenheimer P, Niederer P (2008) Diastolic ventricular aspiration: A mechanism supporting the rapid filling phase of the human ventricles. J Theor Biol 250: 581–592. 18068727 10.1016/j.jtbi.2007.10.033

[31] Zhong L, Ghista D, NG E, Chua T, Lee CN, et al (2007) Left ventricular functional indices based on the left ventricular elastances and shape factor. Journal of Mechanics in Medicine and Biology 07: 107–116.

[32] Watanabe S, Shite J, Takaoka H, Shinke T, Imuro Y, et al (2006) Myocardial stiffness is an important determinant of the plasma brain natriuretic peptide concentration in patients with both diastolic and systolic heart failure. European heart journal 27: 832–838. 16464912 10.1093/eurheartj/ehi772

[33] Venugopal JR, Prabhakaran MP, Mukherjee S, Ravichandran R, Dan K, et al (2012) Biomaterial strategies for alleviation of myocardial infarction. J R Soc Interface 9: 1–19. 21900319 10.1098/rsif.2011.0301 PMC3223634

[34] Hassan M, Hamdi M, Noma A (2012) The nonlinear elastic and viscoelastic passive properties of left ventricular papillary muscle of a Guinea pig heart. Journal of the Mechanical Behavior of Biomedical Materials 5: 99–109. 22100084 10.1016/j.jmbbm.2011.08.011

[35] Bagnoli P, Malagutti N, Gastaldi D, Marcelli E, Lui E, et al (2011) Computational finite element model of cardiac torsion. Int J Artif Organs 34: 44–53. 21298621 10.5301/ijao.2011.6313

[36] Le Rolle V, Hernández AI, Richard P-Y, Donal E, Carrault G (2008) Model-based analysis of myocardial strain data acquired by tissue Doppler imaging. Artificial Intelligence in Medicine 44: 201–219. 18722757 10.1016/j.artmed.2008.06.001

[37] Niederer SA, Smith NP (2009) The role of the Frank–Starling law in the transduction of cellular work to whole organ pump function: A computational modeling analysis. PLoS computational biology 5: e1000371. 19390615 10.1371/journal.pcbi.1000371 PMC2668184

[38] Evangelista A, Nardinocchi P, Puddu P, Teresi L, Torromeo C, et al (2011) Torsion of the human left ventricle: Experimental analysis and computational modeling. Progress in biophysics and molecular biology 107: 112–121. 21791224 10.1016/j.pbiomolbio.2011.07.008

[39] Kerckhoffs RC, Omens JH, McCulloch AD (2012) A single strain-based growth law predicts concentric and eccentric cardiac growth during pressure and volume overload. Mechanics research communications 42: 40–50. 22639476 10.1016/j.mechrescom.2011.11.004 PMC3358801

[40] Kroon W, Delhaas T, Arts T, Bovendeerd P (2007) Constitutive Modeling of Cardiac Tissue Growth. In: Sachse F, Seemann G, editors. Lect Notes Comput Sc: Springer Berlin Heidelberg. 340–349.

[41] Eriksson T, Prassl A, Plank G, Holzapfel G (2013) Influence of myocardial fiber/sheet orientations on left ventricular mechanical contraction. Mathematics and Mechanics of Solids.

[42] Lee LC, Wenk JF, Zhong L, Klepach D, Zhang Z, et al.. (2013) Analysis of Patient-specific Surgical Ventricular Restoration-Importance of an Ellipsoidal Left Ventricular Geometry for Diastolic and Systolic Function. Journal of Applied Physiology. 10.1152/japplphysiol.00662.2012 PMC3727014 23640586

[43] Martina JR, Bovendeerd PH, de Jonge N, de Mol BA, Lahpor JR, et al.. (2013) Simulation of Changes in Myocardial Tissue Properties During Left Ventricular Assistance With a Rotary Blood Pump. Artificial organs. 10.1111/j.1525-1594.2012.01548.x 23278527

[44] Teske AJ, De Boeck B, Melman PG, Sieswerda GT, Doevendans PA, et al (2007) Echocardiographic quantification of myocardial function using tissue deformation imaging, a guide to image acquisition and analysis using tissue Doppler and speckle tracking. Cardiovasc Ultrasound 5: 27. 17760964 10.1186/1476-7120-5-27 PMC2000459

[45] Masugata H, Mizushige K, Kinoshita A, Sakamoto S, Matsuo H, et al (2000) Comparison of left ventricular diastolic filling with myocyte bulk modulus using doppler echocardiography and acoustic microscopy in pressure-overload left ventricular hypertrophy and cardiac amyloidosis. Clinical cardiology 23: 115–122. 10676603 10.1002/clc.4960230209 PMC6655005

[46] Kolipaka A, Araoz PA, McGee KP, Manduca A, Ehman RL (2010) Magnetic resonance elastography as a method for the assessment of effective myocardial stiffness throughout the cardiac cycle. Magnetic Resonance in Medicine 64: 862–870. 20578052 10.1002/mrm.22467 PMC3035166

[47] Klabunde RE (2011) Cardiovascular physiology concepts: Wolters Kluwer Health.

[48] Courneya CAM, Parker MJ (2010) Cardiovascular Physiology: A Clinical Approach [With Access Code]: Wolters Kluwer Health.

[49] Stouffer G (2011) Cardiovascular hemodynamics for the clinician: Wiley. com.

